# Evolutionary interplay between structure, energy and epistasis in the coat protein of the *ϕX*174 phage family

**DOI:** 10.1098/rsif.2016.0139

**Published:** 2017-01

**Authors:** Rodrigo A. F. Redondo, Harold P. de Vladar, Tomasz Włodarski, Jonathan P. Bollback

**Affiliations:** 1 IST Austria, Am Campus 1, 3400 Klosterneuburg, Austria; 2 Center for the Conceptual Foundations of Science, Parmenides Foundation, 82049 Pullach, Germany; 3 Department of Structural and Molecular Biology, University College London, London WC1E 6BT, UK

**Keywords:** phylogenetics, ancestral reconstruction, structure prediction, experimental evolution, stabilizing selection, high-order epistasis

## Abstract

Viral capsids are structurally constrained by interactions among the amino acids (AAs) of their constituent proteins. Therefore, epistasis is expected to evolve among physically interacting sites and to influence the rates of substitution. To study the evolution of epistasis, we focused on the major structural protein of the *ϕ*X174 phage family by first reconstructing the ancestral protein sequences of 18 species using a Bayesian statistical framework. The inferred ancestral reconstruction differed at eight AAs, for a total of 256 possible ancestral haplotypes. For each ancestral haplotype and the extant species, we estimated, *in silico*, the distribution of free energies and epistasis of the capsid structure. We found that free energy has not significantly increased but epistasis has. We decomposed epistasis up to fifth order and found that higher-order epistasis sometimes compensates pairwise interactions making the free energy seem additive. The d*N*/d*S* ratio is low, suggesting strong purifying selection, and that structure is under stabilizing selection. We synthesized phages carrying ancestral haplotypes of the coat protein gene and measured their fitness experimentally. Our findings indicate that stabilizing mutations can have higher fitness, and that fitness optima do not necessarily coincide with energy minima.

## 1. Introduction

A central question in ‘evolutionary biochemistry’ [1] is how the structure and function of proteins determine their evolution (see reviews in [2,3]). While the traditional approach using phylogenetics allows detection of signatures of selection at the amino acid (AA) and nucleotide level, the specific causes for the observed and inferred genetic diversity in protein sequence, structure and function, often remains unknown [2,4]. Phylogenetics incorporates very little biochemical and structural information and thus has been criticized [2,5]. This gap is in part, due to the complexity of the factors determining macromolecular structure and to the uncertainty of what selection is acting on. Even when signatures of selection are evident in a phylogeny, it can act on complex genotype–phenotype maps, favouring not specific AAs at specific sites, but complete traits encoded by multiple AAs within and between proteins in a non-additive way (i.e. epistatic effects). Moreover, the structure, conformational constraints, kinetics and folding dynamics of macromolecules are important components for their function and of organismic fitness [6–9]. This multifaceted problem calls for combined approaches between evolutionary and structural biology [2].

The aim of this work is to understand the evolution of the capsid of the bacteriophage *ϕ*X174 group (Microviridae). In this family, the capsid is made up of four proteins: the coat, scaffold, major and minor spike proteins. The capsid is structurally conserved across species of Microviridae; the sequence variation in the coat protein is low (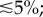 divergence). We specifically focus on the coat protein, which is the central structural constituent of the Microviridae capsid and study two main aspects: the distribution of epistatic effects and selection. We estimate the phylogeny of this group and reconstruct the ancestral states for each AA at every internal node. From these data, we determine which haplotypes to model, computationally determine their free energy and epistasis and experimentally synthesize some of them to assay their fitness effects.

Two actual questions regarding protein structure are: (i) whether function and structure are close to a fitness optimum and (ii) how this relates to a sequence coding for a structure at energetic minimum. We approach the problem combining methods from evolutionary genetics and from computational structural biology to better understand the evolution of free energies of the coat protein, study the distribution of high-order epistasis and infer a fitness landscape, which allows the value of the optimum to be determined. However, we find that this evolutionary point does not correspond to a haplotype that is at an energetic minimum.

Prior experimental works using site-directed or random mutagenesis have shown that while most substitutions result in a decrease of stability, a significant minority can increase it [10–14]. Altogether, this is indicative that most evolved sequences are close, but not exactly, at an energetic minimum. We find exactly this pattern, which can be interpreted as populations maintaining fitness load due to mutation and drift.

By viewing the free energy of the capsid as an evolvable trait, we ask how interactions among AAs give rise to epistasis. Free energy is the capacity to do work, which here refers to unfolding, resulting in structural changes of the protein. Being fully determined by the physical basis of the structure and its environment, free energy is sequence-dependent. Therefore, variability in sequences will result in a distribution of free energies. If the genetic composition changes through the phylogeny, so does the distribution of free energies.

Through molecular analysis, biophysical calculations and experimental essays, we address the interplay between epistasis, selection and molecular evolutionary rates. This connection between structural biology and evolution has direct implications for the importance of structure–function relationships and the role of epistasis in molecular evolution [5–7,15,16].

## 2. Results

### 2.1. Phylogeny and ancestral reconstruction

**Figure 1.**
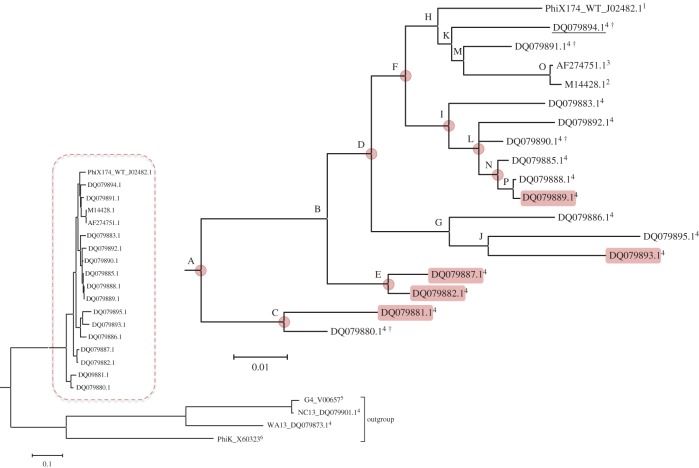
Phylogenetic tree of the coat protein of the *ϕ*X174 and related phages used in this study. 1: Sanger *et al*. [17], 2: Lau & Spencer [18], 3: Wichman *et al*. [19], 4: Rokyta *et al*. [20], 5: Godson *et al*. [21], 6: Kodaira *et al*. [22]. ^†^Extant species with a coat protein identical to an ancestral haplotype. The underlined species has a coat protein identical to the consensus of extant sequences. Nodes marked from A to P represent the internal node for which the ancestral reconstructions were performed, see also table 1. Highlighted extant species and nodes indicate the presence of epistatic interactions. (Online version in colour.)

**Figure 2.**
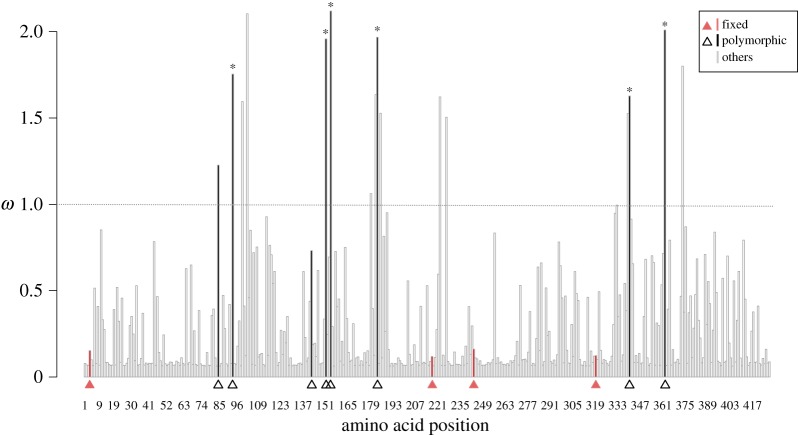
Distribution of *ω*(d*N*/d*S*) per AA site of the *ϕ*X174 coat protein estimated by PAML, using the model M8 and the consensus tree from the Bayesian analyses (figure 1). The mean is 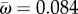. Filled triangles ancestrally fixed sites; open triangles: ancestral uncertain sites. Asterisks denote ancestral uncertain sites with statistically significant *ω*. (Online version in colour.)

Figure 1 shows the Bayesian phylogenetic reconstruction of the coat protein of *ϕ*X174 using a codon model of substitution. The average nucleotide divergence among the ingroup *ϕ*X174 sequences was 0.047, with a low average d*N*/d*S* ratio *ω* = 0.060 (95% credible interval: 0.049, 0.092) and a maximum-likelihood estimate of *ω* = 0.084, consistent with strong purifying selection (figure 2).

**Table 1.** Uncertain sites found in the ancestral reconstructions of each internal node of the phylogenetic tree (figure 1). Node A is the most recent common ancestor of the ingroup *ϕ*X174 species analysed and presents the eight ancestral alternatives discussed in the text, ART is ktpeqqsa. The number of haplotypes and the most likely haplotype for each node with the posterior probability associated is also presented. Capital letters represents uncertain sites and lower letters fixed sites in that node.

node	uncertain site								no. haplo.	most likely haplotypes	Pr.
A	K83Q	T92S	P141A	E150Q	Q153E	Q182L	S339A	A361V	256	KSPEEQAA	0.067
B	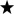	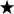	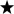				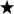		16	KSAeeqSa	0.81
C									1(2)^a^	qsaeelaa	0.97^a^
D	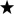	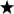		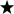			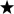		16	KTpEeqAa	0.43
E		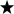 /n.a.^b^							4	kNpqeqaa	0.46
F		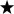		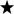			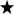		8	kTpEeqSa	0.73^c^
G		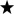	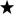	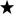	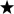			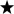	32	kTPEEqsA	0.68^c^
H					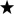				2	ktpeEqsa	0.89^c^
I				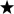	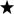		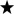		8(16)^a^	ktpEEqAa	0.424^a^
J		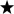						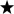	4	kTaeeqsA	0.76
K									1	ktpeeqsa	0.99^c^
L				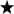	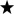				4(8)^a^	ktpEEqaa	0.64^a^
M		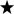							2	kTpeeqsa	0.98^c^
N				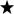					2(4)^a^	ktpEqqaa	0.51^a^
O									1	ktpeeqsv	0.99
P									1	ktpqqqaa	0.99

^a^Nodes with one additional uncertain site (G101R) not present in the ingroup ancestral Node A.

^b^Node E showed multiple alleles on site T92S/n.a.

^c^Nodes whose most likely haplotype is identical to the consensus of the extant species.

The ancestral reconstruction for the ingroup *ϕ*X174 has 34 nucleotide differences (12 non-synonymous and 22 synonymous) relative to the Sanger strain (SS) of the *ϕ*X174 phage (GeneBank accession J02482). As our goal is to understand the role of energetic changes in the major coat protein, we focus solely on AA substitutions (table 1), for which we carried out a separate phylogenetic analysis (electronic supplementary material, S1).

At the ancestral node, eight out of 12 AA changes occur as two different (uncertain) alleles. All possible allelic combinations give 256 putative ancestral haplotypes. The four remaining positions (3 V, 216R, 242F and 318R) are fixed in the phylogeny except in the SS, and will be termed ‘ancestrally fixed’.

The ancestral haplotype containing these four fixed positions plus the remaining eight in the same state as the SS is dubbed Ancestral Reference Type (ART; table 1). We chose to use ART as a reference to minimize methodological biases relative to structural analyses (Material and methods). The ancestral haplotype containing all eight uncertain alleles in a state different from ART is called AT_8_. All reported ancestral probabilities are for the AA reconstructions. AA substitutions are presented relative to ART.

Each ancestral haplotype has a posterior probability of being the true ancestor. In our case, the most likely ancestral haplotype (Pr = 0.067) has only three differences from ART (T92S, Q153E and S339A; table 1), more likely than the uninformative prior probability, 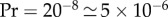.

Figure 2 shows that nearly all of the sites in the coat protein have low *ω* values indicating purifying selection [23]. Six out of the eight variable sites in the ancestral node are under diversifying selection, indicated by *ω* > 1 (figure 2). The consensus of extant sequences coincides with the ancestral haplotype Q153E, which also corresponds to the AA sequence of species DQ079894.1. Three other ancestral haplotypes are also present in the extant species: DQ079890.1, DQ079891.1 and DQ079880.1 (figure 1).

### 2.2. Spatial location of the ancestral haplotypes

**Figure 3.**
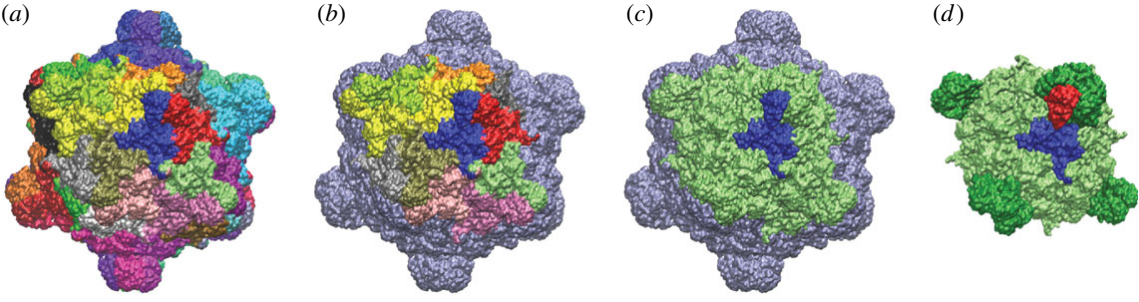
Capsid structure and molecular model of the fragment. Molecular model of the *ϕ*X174 capsid (PDB:2BPA) highlighting (*a*) the repetitive constituting protein subunits (each colour is a subunit), (*b*) the 12 protein subunits in the fragment employed for our calculations, (*c*) the fragment and one protein subunit and (*d*) the fragment showing one coat protein (blue) and one spike protein (red).

**Figure 4.**
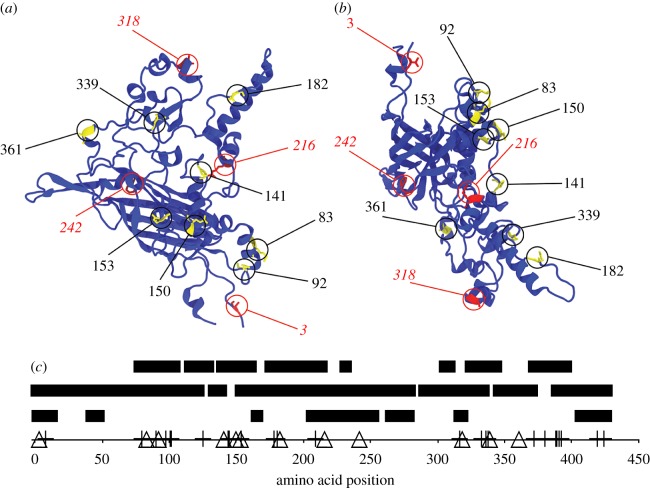
Coat protein and spatial location of the variable sites. (*a*) Top view (from the solvent's perspective). (*b*) Side view. Residues in yellow, labelled by black bold upright fonts: uncertain sites. Residues in red, labelled by red italics regular fonts: ancestrally fixed sites. (*c*) Classification of the position of the AAs according whether they are (top bars) exposed to the solvent, (middle bar) at the interface between proteins or (lower bar) exposed to the internal space of the capsid. Crosses: variable sites in the extant species; triangles: ancestral variable sites.

Figure 3 shows the crystal structure of the SS *ϕ*X174 (PDB:2BPA) and details the fragment employed for structural simulations. Figure 4*a,b* shows the position of the ancestral uncertain sites in the coat protein. Most of these sites face the external milieux, suggestive of relaxed structural constraints (figure 4*c*). The ancestrally fixed sites mostly face the inside.

### 2.3. Free energy at the ancestral state and in extant species

We calculated the free energy of all probable haplotypes for all internal nodes of our phylogeny and extant isolates using FoldX, and with Rosetta only the 256 ancestral haplotypes (due to computational limitation, see Material and methods). These computational structural biology tools allow the estimation of changes in ΔΔ*G* of the haplotypes relative to a reference structure (Material and methods). We chose ART as the reference, because it has the least changes (four AAs) from the known structure of SS. By comparing the free energy of haplotypes to an evolutionary equidistant reference point at the ancestral state, and not to an extant leaf (e.g. the SS), we avoid potential biases in our analyses. The ΔΔ*G* calculated with FoldX and Rosetta will be denoted by subscripts FX and R, respectively. Owing to computational constraints, we chiefly employ the FoldX simulation dataset, but to the extent of our possibilities we support our findings with Rosetta's simulation results.

### 2.4. The energy spectrum of *in silico* random substitutions is wide

**Figure 5.**
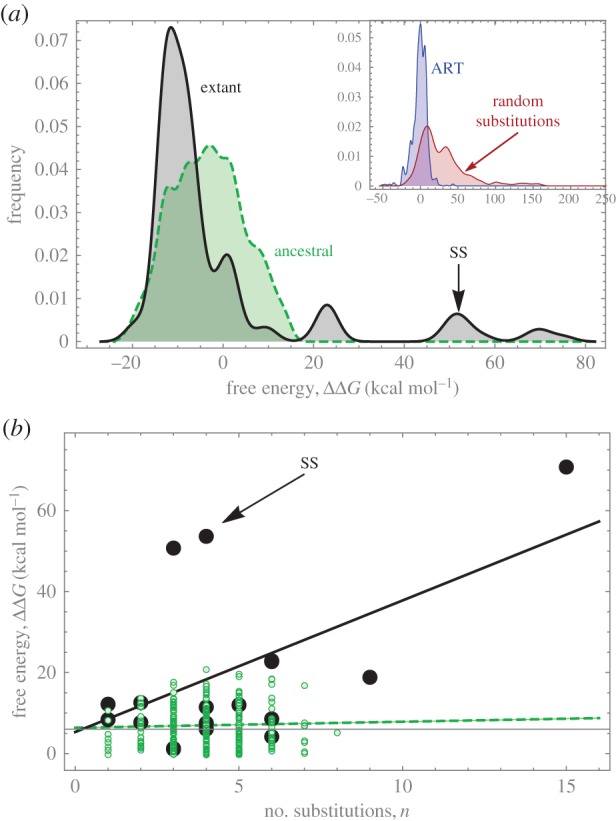
(*a*) Histogram of free energies relative to ART. Black solid line, extant species (mean = −3.36 kcal mol^−1^); green dotted line, ancestral haplotypes (mean = −1.38 kcal mol^−1^). Inset: histogram of 127 random mutants having between 1 and 5 substitutions, compared to SS. (*b*) Free energy (relative to ART) versus the number of AA substitutions (*n*). Black large bullets, extant species; green small bullets, ancestral haplotypes. Lines are linear regression: for the ancestrals (dotted green line), ΔΔ*G* = 6.39 + 0.15*n* kcal mol^−1^ (*p* = 0.5); for the extant species (black line), ΔΔ*G* = 5.32 + 3.25*n* kcal mol^−1^ (*p* = 0.024). All values from FoldX. (Online version in colour.)

Random *in silico* mutations of the capsid protein results in a free energy distribution that is much wider than that of the ancestral or extant species (figure 5*a*). While the maximum ΔΔ*G* of the ancestral haplotypes is approximately 15 kcal mol^−1^, in the random substitutions it is approximately 264 kcal mol^−1^. Both of these extreme values occur with only four substitutions. The smaller variance of the ancestral distribution is consistent with purifying selection acting on the capsid, assuming that mutations resulting in large ΔΔ*G* are unfit.

### 2.5. Average increase of free energy with increasing substitutions

We performed a linear regression in order to test whether ΔΔ*G* increases with the number of substitutions. In the ancestral set, there is no significant trend (FoldX: slope = 0.15 kcal mol^−1^, ANOVA *p* = 0.50; figure 5*b*. Rosetta: slope = 0.03, ANOVA *p* = 0.40). This is expected when considering that, among the 256 putative ancestors, ART is an arbitrary reference and from it there should not be any particular trend in energetic states.

In the extant haplotypes, there is a significant trend where each substitution adds on average 3.25 kcal mol^−1^ (ANOVA *p* = 0.024; FoldX. No calculations with Rosetta). This slope is consistent with the mean value of the distribution of mutational effects. The higher slope in the extants is expected because mutations accumulate as lineages diverge. Note that the slope is heavily driven by three points which have notably high free energies (DQ079885, SS and DQ079892; the latter having 15 substitutions).

### 2.6. Mutational effects have a positively skewed distribution

**Figure 6.**
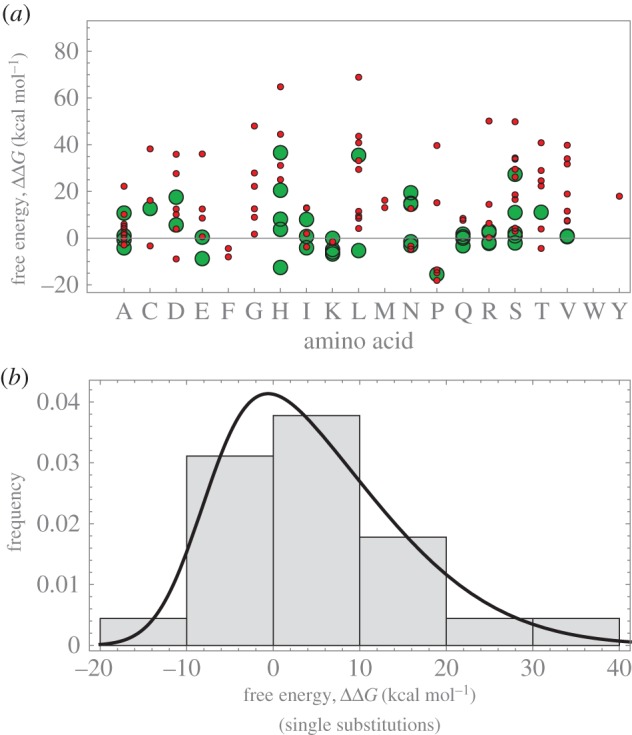
(*a*) Energy contributions of the different AAs. Large bullets, single substitutions occurring in the alignment; small bullets, single substitutions of a randomly generated dataset. (*b*) Grey bars: distribution of single substitution effects. Solid curve: skew-normal distribution with location, scale and shape parameters –7.84, 16.49, 3.57 (maximum-likelihood estimators), respectively, (mean = 4.83, variance = 111.44 and skewness = 0.74). Results from FoldX dataset. (Online version in colour.)

Following the terminology of quantitative genetics ([24], p. 122; see also [25,26]) free energy differences of single substitutions are called ‘additive effects’. These effects follow a skew-normal distribution with mean of 4.83 kcal mol^−1^ per AA (figure 6*b*) and positive skew, arguably suggesting the capsid being close to (but not exactly at) an energetic minimum [3]. While effects can be as small as 0.14 kcal mol^−1^ (in absolute value), others can be as high as 36.77 kcal mol^−1^. This distribution shape is consistent with that of quantitative traits, which also show positive skews [27,28].

### 2.7. Few substitutions drive free energy changes

Relative to ART, the SS's coat protein has a large ΔΔ*G*_FX_ *=* 53.79 kcal mol^−1^ (*p* ∼ 10^−8^). Two substitutions, R216H and F242 L, explain 56.34 kcal mol^−1^ of it; the two remaining substitutions (V3I, A318 V) contribute by –3.07 kcal mol^−1^. Although these four substitutions conserve charge and hydrophobicity, the former two involve aromatic rings, introducing significant steric reconfigurations, packing density and changes of electrostatic interactions.

Species DQ079885 also has a large deviation (ΔΔ*G*_FX_ = 50.88 kcal mol^−1^); two substitutions (D338H and E145D) add 54.52 kcal mol^−1^ (note the common presence of histidine); the third substitution, S339A reduces it by 4 kcal mol^−1^. Of the 15 AA differences in species DQ079892, three (Y102S, T144N and V333I) contribute with 62.30 kcal mol^−1^ (88%) of the ΔΔ*G*_FX_ = 70.90 kcal mol^−1^. The remaining 12 additively contribute the remaining 12%. The free energy differences of every single substitution varies according to two factors: the original and derived AAs, and the position (surface or buried) in which these occur. Notably, histidine and leucine tend to have the strongest effects (figure 6*a*).

From these examples, we conclude that most free energy deviations are driven by a few substitutions of large effect, again coincident with the pattern of substitutions in quantitative traits (figure 6*b*). In the discussion, we address biophysical factors for this pattern.

### 2.8. Strength and causes of epistasis

Structural epistasis is calculated by subtracting the additive free energy of the constituting single substitutions from the free energy of that haplotype. We consider epistasis 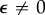 when the *p*-value of a *t*-test is below 
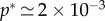
. This test was performed only with FoldX (Material and methods and electronic supplementary material, S2).

About 37% of the multiple mutants are epistatic: 66 in the ancestral set, two in internal nodes (one in Node C—K83Q, T92S, G101R, P141A, Q153E, Q182 L, S339A and E150Q, Q153E, S339A, also shared among several ancestral nodes), and five in extant species (DQ079881, DQ079882, DQ079887, DQ079891, DQ079893; figure 1).

**Figure 7.**
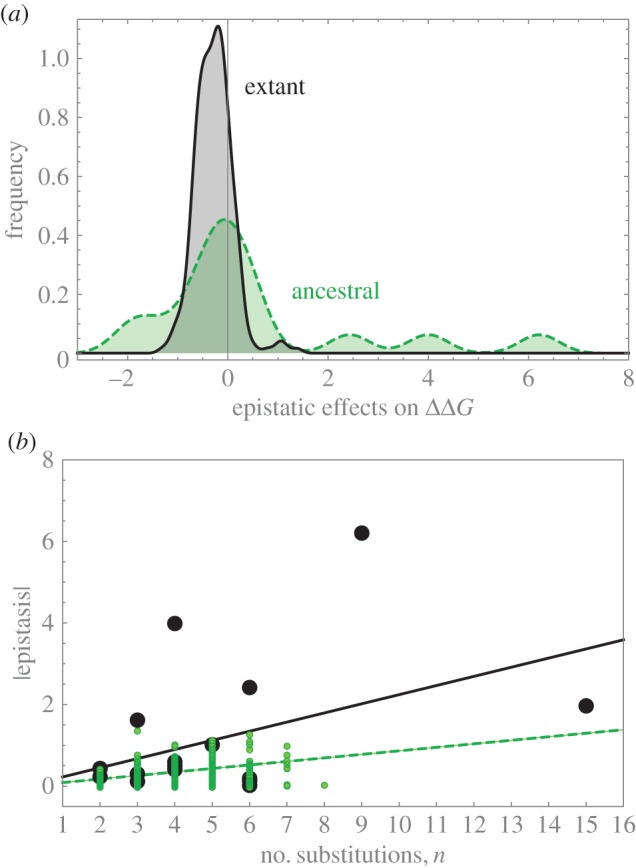
(*a*) Histograms of epistatic values. Solid line: extant species (mean = 0.48 kcal mol^−1^; variance = 4.39); dashed line: ancestral haplotypes (mean =−0.28 kcal mol^−1^; variance = 0.14). (*b*) Relationship between epistasis and the number of AA substitutions (*n*). Large bullets, extant species; small bullets, ancestral haplotypes. The lines are linear regressions. For the ancestrals (dashed line): 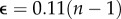 kcal mol^−1^; for the extant species (solid line): 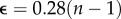 kcal mol^−1^. Results from FoldX dataset. (Online version in colour.)

The distribution of epistatic effects estimated using FoldX's data has a variance of 0.13, while using Rosetta the variance is much larger, 1.8. However, their means are statistically similar (−0.27 and −0.13, respectively; *t*-test on mean equivalence, *p* = 0.30. See the electronic supplementary material, S3 for details). A linear regression of epistasis from FoldX's data on the number of substitutions has a slope of 0.11 kcal mol^−1^ in the ancestral species and a slope of 0.3 with Rosetta's data. Electronic supplementary material, S3 describes and reports bootstrap tests giving *p* < 10^−4^ for both slopes. In the extant species, the trend is stronger, with a slope of 0.22 kcal mol^−1^ (figure 7*b*; FoldX). Epistasis and ΔΔ*G*_FX_ are weakly correlated in the extant species (no data with Rosetta).

### 2.9. Statistical and structural epistasis are correlated but not causally

Epistasis can be physically determined by interactions among AA side chains [29]. This mechanistic source differs from the canonical statistical definition [30]. Using an independent dataset of the 256 ancestors' energy obtained with FoldX, we tested five ANOVA models (additive and up to five-way mixed effects; Material and methods). Akaike's information criterion favours five-way interactions. However, only 18 interaction terms (out of 218) are significant (confidence = 0.050). Statistical epistasis correlates with structural epistasis (electronic supplementary material, S2), even when employing the pairwise model (data not shown). In both the pairwise and five-way models, only five of the 27 pairwise parameters are significant. The pairs with significant epistasis are the same in both models and involve two focal AAs at positions 83 and 153 (inter-AA distance = 33.41 Å). Besides correlating with each other, 83 correlates with 141 (13.47 Å) and with 361 (40.1 Å), and 153 correlates with 150 (42.86 Å) and 361 (13.04 Å). An important observation is that none of these pairs show significant structural epistasis. Moreover, there is no over-representation of these substitutions in epistatic multiple mutants. This raises the question of how meaningful is the interpretation of regression coefficients in association analyses in terms of causal factors.

### 2.10. High-order epistasis cannot always be decomposed into pairwise epistasis

**Figure 8.**
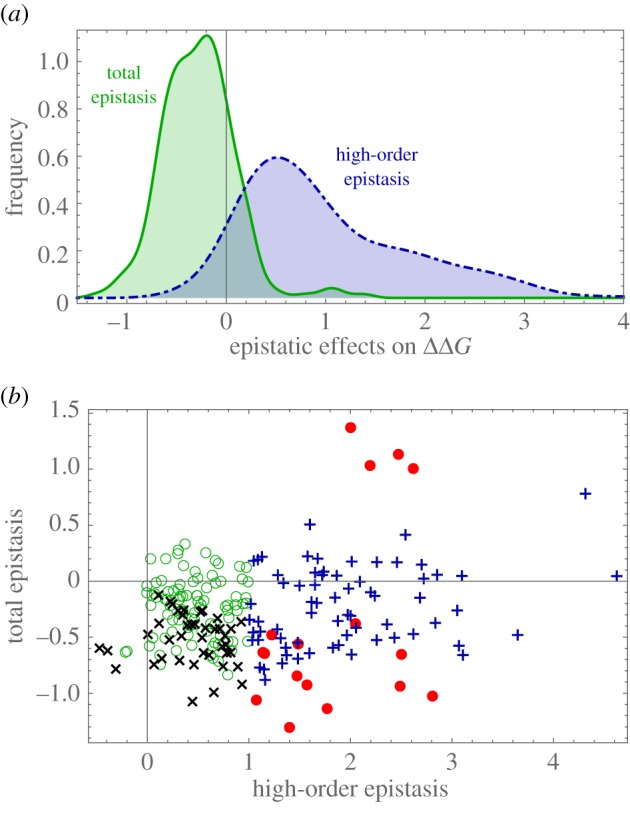
(*a*) Histograms of total and high-order structural epistasis in the ancestors. Solid green line, total epistasis (same as figure 7*a*, shown for reference); dot-dashed blue line, high-order epistasis (mean = 1.024 kcal mol^−1^; variance = 0.77). (*b*) High-order versus total structural epistasis. Green rings, no epistasis; red bullets, total and high-order epistasis; black cross symbols, total epistasis but no high-order epistasis; blue plus symbols, no total epistasis but with high-order epistasis. Results from FoldX dataset.

Pairwise and five-way statistical epistasis are strongly correlated (slope = 0.70, 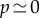, *R*^2^ = 0.86, corr = 0.95), suggesting that pairwise factors dominate interactions. Although pairwise effects are pervasive, there is statistical support for high-order epistasis. The mean value of high-order epistasis is 1.024 kcal mol^−1^ (significantly different than zero; sign test for the median, 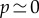; figure 8*a*), and its variance is 0.77 (significantly larger than that of total epistasis = 0.14). In the electronic supplementary material, S3, we present equivalent results with Rosetta.

Using the data from FoldX, we find that compensatory pairwise effects can be of contrary sign to the value of higher-order interactions. It is striking that in some cases these two terms balance each other, resulting in energy values that seem to be additive, but have significant epistasis (blue cross symbols in figure 8).

### 2.11. Fitness assays

Of 10 synthetic constructs containing ancestral versions of the coat protein gene (the eight ancestral uncertain sites, ART and AT_8_), all but one (E150Q) were recovered. Recovered haplotypes presented the same plaque morphology as the SS. Absolute fitness was measured as the growth rate per hour (Material and methods), and relative fitness was computed relative to both ART and SS.

**Figure 9.**
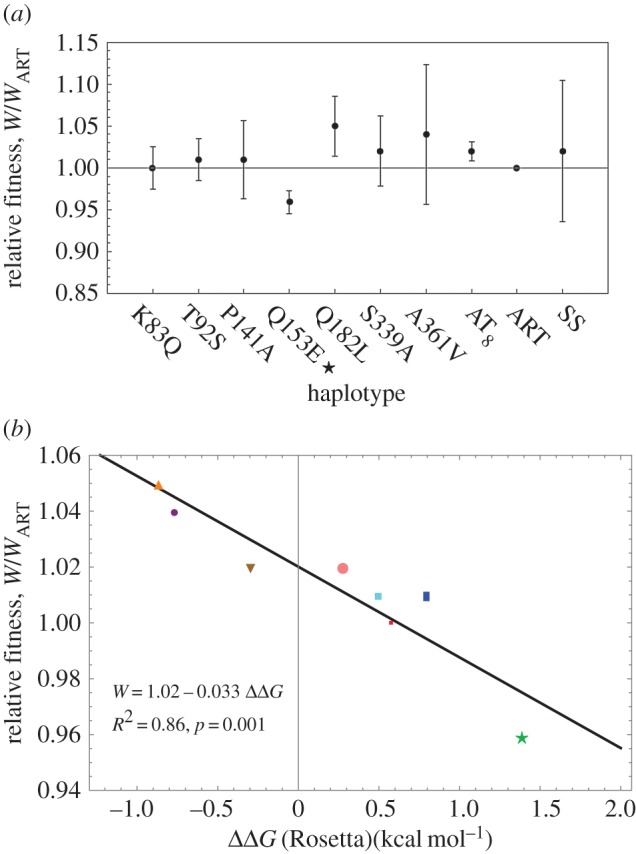
(*a*) Experimental fitness measures (relative to ART). Bars: 1 s.d. on each side. None of the haplotypes have a fitness significantly different from the SS. Star denotes consensus of the extant species. (*b*) Relationship between relative fitness and ΔΔ*G*_R_. Small red square, K83Q; magenta square, T92S; blue rectangle, P141A; green star, Q153E (consensus); orange upright triangle, Q182L; brown downwards triangle, S339A; small purple bullet, A361V; large pink bullet, AT_8_. (Online version in colour.)

As with the free energy calculations, we report fitness relative to ART (figure 9*a*). Relative fitness values were not statistically different from 1, i.e. equal in fitness to ART or SS (*t*-test for mean). The lowest relative fitness corresponds to the ancestor Q153E; note that this is the consensus of extant species. However, fitness measurements are subject to high experimental variance and due to limited data, biologically meaningful fitness effects are hard to resolve. Our average fitness measures are at most 5%, with a relative error between 1.1% and 8.3%, hardly detectable through a *t*-test. Nevertheless, we find a significant trend between relative fitness and ΔΔ*G_R_* (figure 9*b*). (The trend with FoldX is inaccurate; see the electronic supplementary material, S4.) According to this regression, the haplotype that was not recovered (E150Q) has a ΔΔ*G* = −1.214 and would have had the highest relative fitness = 1.06.

## 3. Discussion

### 3.1. Thermal stability versus steric compatibility

It has been observed that the consensus of a group of sequences shows higher thermal stability [31–33] and higher fitness [34,35] than other sequences. The consensus sequence is expected to be at an optimum value because most substitutions tend to be detrimental and, under low mutation rates, derived alleles are not represented in the consensus. However, our results are in contrast to this because the consensus sequence has the lowest fitness and the highest ΔΔ*G* of the assayed haplotypes (figure 9).

We found no evidence for selective preference of substitutions decreasing ΔΔ*G*. There are at least two alternative explanations for this. First, it might be that stability is not severely affected during the evolution of the capsid of *ϕ*X174. If stability would be selected, we would, instead, observe an overall decrease of free energy along the phylogeny. Alternatively, if the capsid is at an energetic minimum, there would not be any substitutions in the mutational neighbourhood that would allow for further decreases of ΔΔ*G*. Which of these two (or other) alternatives hold remains open.

The interpretation that free energy measures stability requires clarification. Assessing stability of the capsid requires energy evaluations not only of the actual configuration but also of alternative states such as when the capsid is unassembled. In addition, we still require knowledge regarding the activation energy for disassembling the capsid. While free energy differences dictate the preferred direction of the conformational change, activation energy dictates the expected waiting time for this change to happen. By contrast, FoldX and Rosetta measure ΔΔ*G* on the structural degrees of freedom of side chains, which do not determine thermostability (cf. [14], for an alternative approach that does not rely on structural calculations). However, it remains true, and an important point, that substitutions that do not affect the native structure (as ours, occurring at the surface; [36]) might significantly affect the folding rates [13], but this is not reflected on FoldX's or Rosetta's calculations.

### 3.2. Distribution of mutational effects

Quantitative trait loci and genome-wide association studies have revealed that additive effects follow right skewed distributions [37,38], observations supported by compelling theoretical arguments [27,28]. Despite the biophysical nature of our trait, the additive effects on free energy have a skew-normal distribution, consistent with quantitative genetics.

An argument explaining skewed distributions is that substitutions of large effect are initially selected since they bring the trait closer to a fitness optimum. Once close to an optimum, only substitutions of small effects allow fine-tuning of the trait to match the optimum trait value. Consequently, there are few opportunities for mutations of large effects to fix, but many for mutations with small effects [16,39]. We observe this factor in our results, supporting evidence for the proximity of the ancestral sequences to an optimum. However, note that, even if this is true, this might only be a local optimum.

Biophysical explanations of the distribution of single substitutions have several causes, some of which follow. First, substitutions in protein surfaces evolve faster than buried AA [36,40] and tentatively have milder effects because solvent-exposed residues have less local interactions with other AAs than buried ones, as indicated by the packing density. This is matched in our data: the eight variable sites in the coat protein are among the ones with the highest *ω*, and are, in fact at the capsid surface. Moreover, on comparing figures 2 and 4*c* a correspondence can be seen between AAs at the surface and values *ω* > 1.

Second, steric effects, where bulky side chains substitute smaller ones, disrupt packing [41], as is the case of His and Leu which have the largest effects and have heavy side chains.

Third, the change in electrostatic potential and van der Waals interactions are naturally dependent on the chemical composition of a focal side chain and its chemical environment [42].

The mechanistic nature behind mutational effects raises the question of whether this distribution remains constant during evolution [43]. Epistasis invariably means that the propensity of AA substitution changes with divergence [5]. But the coat protein of *ϕ*X174 showing only a small degree of divergence, makes this effect harder to detect, even if epistasis is evident. However, we focused on the distribution of effects at the ancestral state and did not compare directly to the expected distributional in extant populations.

### 3.3. Selection

We found evidence that selection is acting on evolution of *ϕ*X174 coat protein, however, the mode of action is still unclear.

The distribution of the effects in the random set is a surrogate for neutrality. This distribution has larger mean and variance than that of the effects along the phylogeny (figure 5). Comparison of both distributions rules out that the latter is neutral [39] (Kolmogorov–Smirnov test 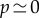, electronic supplementary material, S5), supporting the action of selection.

A first scenario is that directional selection maintains the structure very close to an absolute energetic minimum. Unless there is substantial load, the occurrence of negative mutational effects is unlikely because no substitutions can further diminish the free energy of the capsid. Evidence against this scenario is that the class of mutations that diminish the free energy is not small. Moreover, our experiments show that there are substitutions that decrease free energy and increase fitness (figure 9).

Directional selection with high mutation rates would result in high load, where mutations that decrease free energy should be expected. However, high mutation rates would result in a large spectrum of fixed substitutions causing a higher phylogenetic divergence. The low divergence observed in our data is indicative that selection is much stronger than mutation, which altogether argues against this scenario.

An alternative is that the capsid is under stabilizing selection, where substitutions that deviate the structure from its optimum fitness value are out-selected. As stated before, this fitness optimum does not need to coincide with the energetic minimum. The extant species have a similar energy distribution to the ancestrals. Further evidence comes from simulations with random substitutions: having a wider distribution of effects than the ancestral, it indicates selection. In addition, the low average d*N*/d*S* ratio indicates strong purifying selection, consistent with stabilizing selection.

Another evidence for stabilizing selection is that only 14 sites show a high posterior probability of having *ω* > 1, while 315 sites belong to a category with an extremely low median for *ω* = 0.084. Of the eight ancestral uncertain sites, seven are under positive selection (only one of them has a low posterior probability). This corroborates strong purifying selection, where only few mutations fix along each branch of the phylogeny.

Figure 6 shows that among all substitutions histidine has the strongest effect on ΔΔ*G*. We expect multiple histidine substitutions to result in large free energy deviations. We substituted the four fixed as well as the eight ancestral uncertain sites to histidines, and compared it to 12 substitutions to histidines at random sites (S1H, G57H, F124H, E178H, A198H, G246H, M283H, F291H, G321H, G377H, Q405H, D421H). The latter has ΔΔ*G* = 735 kcal mol^−1^, much larger than the former, with ΔΔ*G* = 213 kcal mol^−1^. This difference is consistent with our hypothesis of purifying (stabilizing) selection because the observed substitutions act at positions that minimize ΔΔ*G* deviations. Many of these substitutions are at buried sites, explaining the stunning difference in ΔΔ*G*.

Although sites that are under positive selection evolve at a higher rate than neutral ones (showing *ω* > 1), the converse is not always true because sites free of functional constraints (e.g. at the surface) may evolve rapidly, even if they are not under positive selection [44]. However, we exclude the latter possibility because our experiments do not show a strong statistical difference in fitness relative to ART or SS. Therefore, we think that the ancestral uncertain sites are not entirely free of constraints. Below we give further thoughts to this.

**Figure 10.**
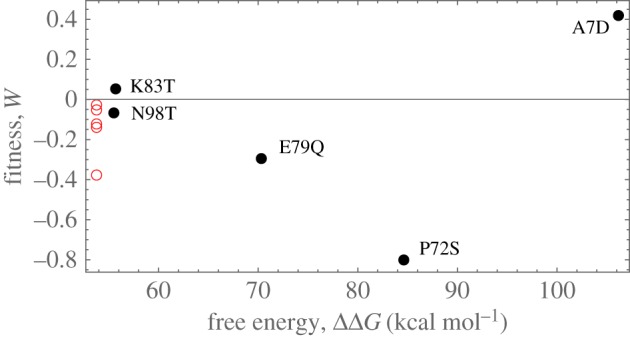
Experimental fitness measures of [45] against ΔΔ*G*_FX_. Black bullets, AA substitutions; redrings, silent DNA substitutions. (Online version in colour.)

Investigations on the distribution of mutational fitness effects of *ϕ*X174 [45] (figure 10) showed that more than two-thirds of 36 random single-point mutations (including synonymous mutations) resulted in fitness changes. Of all substitutions, only five non-synonymous mutations occurred in the coat protein (gene F). Of these, two (K83T and N98T) have no significant effect on fitness, yet both have *ω* > 1 in our phylogeny. The other three changes (A7D, P72S and E79Q) have *ω* < 1 and show a significant effect on fitness, with A7D being beneficial. This pattern is further reflected in a significant negative correlation between fitness effects and *ω* (*R*^2^ = −0.84).

**Figure 11.**
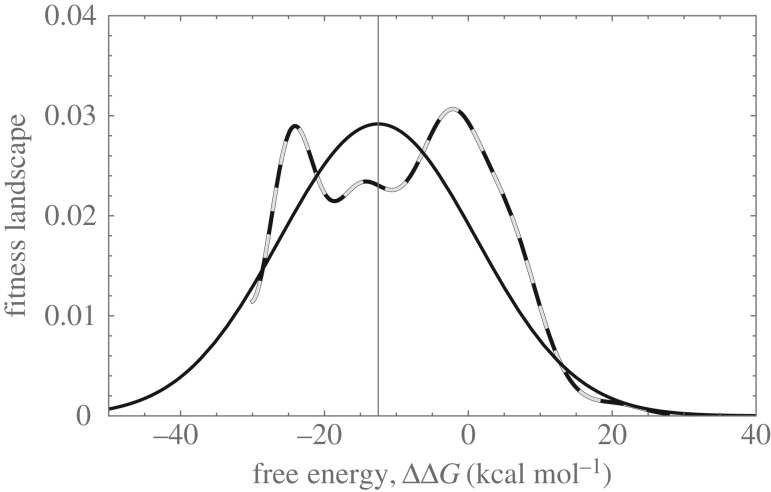
Empirical approximation of a stabilizing selection landscape. Dashed line denotes empirical landscape (using only data for single substitutions in the ancestral node). Black: Gaussian landscape with parameters inferred from the empirical landscape. Note that the scale of the landscape is of arbitrary units, and in this case both functions were normalized. For details on the method of estimating the landscape, see the electronic supplementary material, S5. Results based on FoldX dataset.

Figure 11 presents an estimation of the fitness landscape, employing the distribution of both ancestral haplotypes and random substitutions. We infer that the selective strength is about 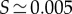, and that the optimum is at 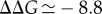 kcal mol^−1^. This optimum fitness value coincides with the peak of the distribution of extant species. We stress that we did not include the extant species in the fitness estimations. The consensus sequence is 7th in rank closest to the peak (ΔΔ*G* = −8.54 kcal mol^−1^), and the most likely haplotype is ranked 55th (ΔΔ*G* = −11.66 kcal mol^−1^). There is no correlation between this ranking and the probability of the ancestral haplotype.

We conclude that stabilizing selection is acting on the coat protein of *ϕ*X174 capsid. What could be its source? Note that the ancestral variable sites occur at the surface of the protein (figure 4*c*). These are known to have less effect on protein stability than those occurring at protein interfaces or enzymatic active cores [10,36]. (Our random simulations are consistent with this known fact.) However, capsid self-assembly can be seen as a cooperative event, and this cooperativity can be compromised by multiple mutations by affecting rate limiting steps [42]. Multiple mutations can affect the kinetics of this process, even if granting the structure of final capsid remains conserved because some mutations affect folding/unfolding rates rather than native structure [13,46]. Therefore, steric or kinetic constraints prior to pro-capsid assembly might be more limiting than thermal stability of the capsid, impairing the ability of the coat protein to undergo particular functional conformational changes in the capsid self-assembly. If substitutions introduce a significant increase in the free energy, there might be steric constraints due to conformational changes. If the free energy is decreased too much, the protein, besides steric impairement, can be more rigid. The interplay between these two factors is a possible source of stabilizing selection.

### 3.4. Epistasis

The relevance of epistasis has long been sought in protein evolution [42], and remains a current question [3,4,6,47]. In evolution, epistasis facilitates mutation accumulation by masking detrimental energetic deviations [16]. This restricts the possible evolutionary paths through a network of few but interconnected (nearly) neutral changes [48,49]. Figure 7*b* reveals an evolutionary increase of epistasis in the *ϕ*X174 family, while there is no significant increase in free energy, which is consistent with the epistatic network idea.

Population genetics arguments indicate that selection is more likely to favour antagonistic (or negative) epistasis over synergistic (positive) epistasis [50]. Assuming that most mutations are deleterious, epistatic substitutions that compensate fitness have higher fixation probabilities. We find the opposite pattern in our data, a positive correlation between free energy and epistasis (data not shown). This antagonistic epistasis hypothesis assumes that there is a genotype that can match the optimum. In some models, it is also assumed that the distribution of epistatic effects can bring a trait arbitrarily close to the optimum. It might be that neither of these two assumptions hold in our case. If an evolutionary optimum value cannot be attained due to physical constraints and there is a negative deviation from the optimum, positive epistasis can be favoured [51]. This can explain the positive correlation between energy and epistasis. (An alternative explanation would be disruptive selection, but we would expect a bimodal distribution of energetic effects, which is not the case.)

Substitutions change the electrostatic field, which propagates beyond the immediate radius of hydrogen bond interactions. Hence, epistasis is better explained by Coulomb, van der Waals interactions, or entropic changes [29]. We point out that we did not find any relationship between epistasis and the establishment or breaking of hydrogen bonds.

We have a contrasting distribution of epistatic effects from FoldX and from Rosetta. The first attributes roughly 10% of the ΔΔ*G* to epistasis (consistent with [16]), whereas the latter inflates epistasis up to 10 standard deviations (electronic supplementary material, S3).

### 3.5. High-order epistasis can compensate pairwise interactions

Most prior work has only considered pairwise epistasis; the usual assumption is that higher-order terms are of lower effect or even negligible. The distributions of second to fifth-order epistasis have comparable magnitude. Epistatic coefficients are normally distributed, ranging between –1.50 and 1.50 kcal mol^−1^ (electronic supplementary material, S2). Our results imply that analyses based on only second-order epistasis can be severely biased [52]. (Statistical epistasis also supports the occurrence of high-order epistasis.)

Our findings show that high-order structural epistasis can compensate pairwise interactions in such a way that, for some haplotypes, the free energy appears to be largely additive. Does this epistatic masking have any relevance? The answer depends on the context. For predicting the energetic values of a given AA sequence, it makes little difference. However, epistasis is known to mask genetic variation, known as cryptic genetic variance. This is important in the evolutionary context because it confers evolvability to the capsid. Cryptic genetic variance occurs when a given allele damps down the detrimental effects of other substitutions. Consequently, although there might be a certain amount of heterozygosity in the population, there is no genetic variation in the trait. Although in our analyses we do not consider populations, there is evidence for cryptic epistasis, which might be an important mechanism in the diversification of the *ϕ*X174 family.

## 4. Concluding remarks

By jointly considering evolutionary and structural analyses, we have determined the distribution of additive and epistatic factors that have been preferred during the evolution of the capsid of the *ϕ*X174-like phages.

We found no evidence for a pervasive decrease of the free energy during the evolution of the capsid. However, we found an increase in structural epistasis, which is better explained in terms of the evolutionary history, than by thermodynamic arguments. This might be surprising for physicists and unsurprising for biologists. In either case, the joint analysis allowed us to understand the mode of evolution in a way that would not have been possible based only on one perspective [2].

Employing structural simulations allowed us to overcome some limitations associated in the estimation of phenotypic effects. For instance, we found no hierarchy in the order of epistasis: pairwise and multiple-way interactions have comparable strengths. We were also able to determine that different orders of epistasis can compensate each other, buffering mutation accumulation. Higher-order epistasis remains an experimental and theoretical challenge, but structural biology has aided us in this understanding.

As a last remark, we emphasize that our approach allowed estimating an evolutionary optimum value. It is not unthinkable that this fitness optimum is determined by a biophysical energetic minimum. Although both need not coincide, the inferred optimum and the minimal sampled energy are rather close, and the difference could be justified by a mutational load. Yet, we agree that this conclusion would require further scrutiny.

Our work is a proof of principle of how evolution acts on the physical basis of a trait, when a genotype–phenotype map is determined from first principles. What the relationship between free energy of the capsid, function and fitness remains unclear. However, the strong signal of selection on free energy provides compelling evidence of such interconnection, irrespective of how complicated its nature is.

## 5. Material and methods

### 5.1. Estimation of the phylogeny

We retrieved from GenBank 18 sequences of *ϕ*X174 *sensu stricto* and four outgroups: G4, NC13, WA13 and *ϕ*K (figure 1). We limited our dataset to: (i) sequences originated from wild isolates of the phage and (ii) complete genome sequences available. We also included the canonical *ϕ*X174 Sinsheimer/SS (J02482, SS).

The major coat protein gene (gene F) of the sequences was aligned using ClustalW, implemented in MEGA 5.2 [53]. The ingroup sequences have all the same size and are perfectly alignable having only two shared small deletions of nine and three continuous nucleotides (three and one AAs) in the positions 1129–1137 and 1147–1149 of the alignment relative to the outgroup. The resulting alignments were used to reconstruct the phylogenies in MrBayes v. 3.2.2 [54].

Nucleotide, AA and codon models were used in the reconstructions assuming a GTR + *γ* site substitution model (+*ω* in the codon model) with a Dirichlet prior on the substitution rates of the GTR model and unconstrained branch lengths. All other parameters were at their default values [54]. Samples for topology and parameters estimates used two independent runs of four Markov chains (one cold and three heated) for 2 × 10^6^ cycles, sampling every 200 cycles and burn-in of 25% (Codon model ran for 3 × 10^6^ cycles, sampling each 300th). All three phylogenetic reconstructions gave concordant topologies (figure 1; electronic supplementary material, S1).

We used PAML 4.8 [55] to estimate the d*N*/d*S* ratio (*ω*) of gene F, using a number of codon evolution models M0, M2a, M7 and M8 on the consensus tree of the Bayesian phylogeny. The value of *ω* is expected to be 1 if there is no selection on that codon (neutral), *ω* < 1 if the site is under purifying selection, and *ω* > 1 for sites under diversifying (positive) selection. The best-fit model is M8, which uses a discrete *β*-distribution (*k* = 10 classes) to model classes with 0 < *ω* < 1 and one additional class with *ω* > 1.

### 5.2. Ancestral state reconstruction

The ancestral reconstruction for the last internal node (Node A, figure 1) was carried out in MrBayes using the topology of the trees estimated for each dataset (AA, nucleotide and codons). As the codon tree was the best resolved tree (i.e. had the least number of polytomies; electronic supplementary material, S1), we used it as a guide to specify constraints defining the internal nodes and reporting posterior probabilities for the ancestral states on each node. An independent run of MrBayes was performed for each node.

For the ancestral reconstructions, we considered not only the most probable sequence for each node but also, taking advantage of the power of Bayesian inference in handling uncertainty of the posterior probabilities, we generated a set of all possible ancestors for each node. A site was considered fixed in the ancestor node if the posterior probability of a state was Pr > 0.99 and was considered uncertain otherwise. Moreover, we only considered uncertain sites that were in agreement across all the reconstructions.

### 5.3. Molecular model of the capsid

Our model is based on the atomic structure of the bacteriophage *ϕ*X174 capsid previously solved by X-ray crystallography, 3 Å resolution [56], (PDB:2BPA). The virion capsid, of *T* = 1 icosahedral symmetry, consists of a repeat of 60 identical asymmetric units (figure 3), with each subunit constituted by three proteins: the major coat protein (protein F, 426 AA), the major spike protein (protein G, 175 AA) and the DNA binding protein (protein J, 37 AA; figure 3*c*).

To study free energy changes of different haplotypes with FoldX, we modelled a capsid fragment that consists of 12 subunits: one focal coat protein (as well as one DNA binding and one major spike proteins) surrounded by 11 other identical subunits (figure 3). This complex represents one-fifth of the virion capsid and takes into account the influence of neighbouring protein chains that might affect energy through protein–protein interactions.

The 12-subunit structure was optimized by minimizing its energy. We employed the Amber ff99SB-ILDN force field [57] using the GROMACS 4.5 molecular dynamics simulation package [58]. The energy minimization was first executed in vacuum followed by minimization in explicit solvent. This optimized structure was used as a reference and as a starting point for further analyses.

When considering substitutions, all 12 copies of the coat protein in the fragment were mutated to accounting for inter-protein interactions.

Free energy changes computed with Rosetta use only a single copy of the coat protein. This is because Rosetta is more computationally demanding than FoldX.

### 5.4. Energetic analysis

The free energy change was evaluated with the protein design package FoldX (v. 3.0 beta 5.1). FoldX estimates the free energy of unfolding in a given structure relative to another reference structure. Its semi-empirical force field considers a weighted combination of physical and statistical energy terms calibrated to fit experimental ΔΔ*G* values from mutational experiments [59–62].

The reference structure for our calculations was the capsid fragment described above, but we included the ancestrally fixed sites (table 1). As these substitutions appear in all extant species except in the SS, this haplotype (ART) is an appropriate reference structure.

When using FoldX, calculation for each haplotype is for a complete capsid fragment. Thus, we divided the output ΔΔ*G* by 12 to account for energy per copy of the coat protein. Because both models (ART and haplotype structures) have the same interface with the solvent, the contribution of the fragments' interfaces to the ΔΔ*G* cancel out on average, and the only remaining effect of the solvent is on the substitutions.

FoldX might be inaccurate for a large number of substitutions [63]. However, most of our sequences have only few changes justifying its use. Of the 38 polymorphisms in the ingroup, the ancestral has at most 12 and the extant species at most 15.

We tested consistency of our findings with ddg_monomer protocol from Rosetta [64]. This protocol is an alternative method used to predict changes in protein stability (ΔΔ*G*) induced by point mutations, with limited backbone conformational freedom [65]. It requires minimization of the initial structure, which we conducted using a single chain as a starting model (ART). After introducing mutations, ddg_monomer optimizes all side chain rotamers in both ART and mutant structures, these steps follow three rounds of gradient based minimization, which allows small changes in backbone conformation. We set up ddg_monomer protocol as recommended by Kellogg *et al*. [65] with 15 iterations of optimization, due to computational costs. The final ΔΔ*G* values were converted from Rosetta Energy Units to kcal mol^−1^ as previously described [65].

### 5.5. Structural simulations

Capsid fragments simulations ran at 25°C. With FoldX, we consider a statistically representative sample of ΔΔ*G* by calculating at least 20 replicas to account for alternative energetic minima biasing the estimate [63]. Pilot simulations indicated that 15 replicates are sufficient for the sample variance of ΔΔ*G* to be stable. (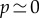 in variance ratio tests between bootstrapped distributions of ΔΔ*G* with 5 and *n* = 10, 15, 20 replicates; no significant difference between 15 and 20 replicas; data not shown).

We generate empirical distributions of free energies for each haplotype in all internal ancestral nodes, for the extant species and for each single substitution occurring in the alignment. (The latter is required to estimate the epistatic effects.)

With Rosetta, we only considered an average value resulting from 15 iterations (not individually reported by the package).

### 5.6. Estimation of structural epistasis

Epistasis is defined as non-additive effects on a trait, in this case ΔΔ*G*. We estimate epistasis 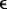 of a haplotype 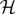 as5.1
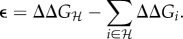


The data from each haplotype and their corresponding single substitutions are not paired. Moreover, different simulations can have different numbers of replicates. Therefore, we estimate the average epistatic value, 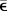. We test statistically whether epistasis is negative, positive or zero by performing a *t*-test using the statistic5.2
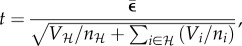
where *V_k_* = var(ΔΔ*G_k_*), is the sample variance of the free energies of the haplotype *k* and *n_k_* is the number of replicates. We employ Welch's approximation for the degrees of freedom for unequal sample sizes.

### 5.7. Estimation of statistical epistasis

Statistical epistasis is based on the model:5.3
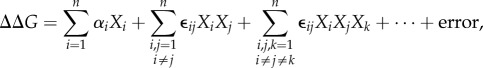
where *α_i_* are additive factors, 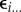 epistatic factors and *X_i_* incidence variables: *X_i_* = 0 for ART allele and *X_i_* = 1 for the alternative allele. Factors *α* and 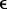 are estimated using an ANOVA. In the design matrix of the ANOVA, we employ all energy points, not their averages (4476 data values). The data limit us to consider epistasis up to five-way interaction. Akaike information criterion was computed to determine model preference.

### 5.8. Experimental methods

To assess the fitness of the haplotypes found in the ancestral reconstructions, we synthesized 10 of the 256 possible ancestor haplotypes, each of the eight single variants (K83Q, T92S, P141A, E150Q, Q153E, Q182 L, S339A and A361 V), ART and the AT_8_.

These synthetic genes were then cloned in the Sinsheimer/SS replacing the gene F and transformed in *Escherichia coli* C strain to obtain phages with capsids containing the ancestral variants (see the electronic supplementary material, S4 for full description of experimental methods).

Absolute fitness was estimated as growth rate of the phages, a measure of population doublings per hour in the presence of excess of bacterial host [66]:5.4
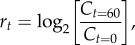
where *C_t_* is the concentration of the phage at measurement time *t*. Then, we estimated relative fitness against a reference (to both ART and SS) by taking the ratio of the absolute fitness of a given haplotype *r_i_* against the absolute fitness of the reference, *r_o_* [66]. Each fitness measurement is based on a total of 16–24 replicates for each ancestral haplotype and 32–48 replicates for SS and ART, with an experimental design that accounts for variance in dilution, plating and handling during the essays (electronic supplementary material, S4).

## Supplementary Material

Phylogenetics and ancestral reconstructions

## Supplementary Material

Statistical methods for estimating epistasis

## Supplementary Material

Calculations and comparisons with Rosetta

## Supplementary Material

Extended description of experimental procedures and results

## Supplementary Material

Inference of the fitness landscape

## Supplementary Material

Data
