# Supplementary material for: Evolutionary interplay between structure, energy and epistasis in the coat protein of the ϕX174 phage family
Source: J R Soc Interface. 2017 Jan;14(126):20160139. doi: 10.1098/rsif.2016.0139 (PMC5310724; doi:10.1098/rsif.2016.0139)
Supplement: Phylogenetics and ancestral reconstructions [file rsif20160139supp1.pdf]

Evolutionary interplay between structure, energy, and epistasis in the coat protein of the $\phi X174$ phage family.

Electronic Supplementary Material 1: Phylogenetics and ancestral reconstructions.

Rodrigo A.F. Redondo, Harold P. de Vladar,
Tomasz Włodarski and Jonathan P. Bollback

Phylogenetic trees for the $\phi X174$ coat protein were inferred using MrBayes using three data frameworks: amino acids (AA), nucleotide and codon datasets. The codon and nucleotide bayesian consensus trees are compatible and almost identical in topology with the nucleotide tree. Besides two additional polytomies, they are otherwise identical clades. The AA tree has a larger number of unresolved branches and one incompatible clade with the codon and nucleotide trees.

However the ancestral reconstruction for the ingroup sequences (Node A in Fig. 1 of the main text) has the same 8 ancestral polymorphisms and 4 fixed ancestral states as in the AA reconstructions. The codon reconstruction has an extra polymorphism at R101G in Node A (although with very low probability) and the A361V substitution is missing. The nucleotide tree has an extra polymorphism in D338H.

Because the codon tree presented the best resolution, we chose it to specify the constraints for the reconstruction of the sequences at the internal nodes.

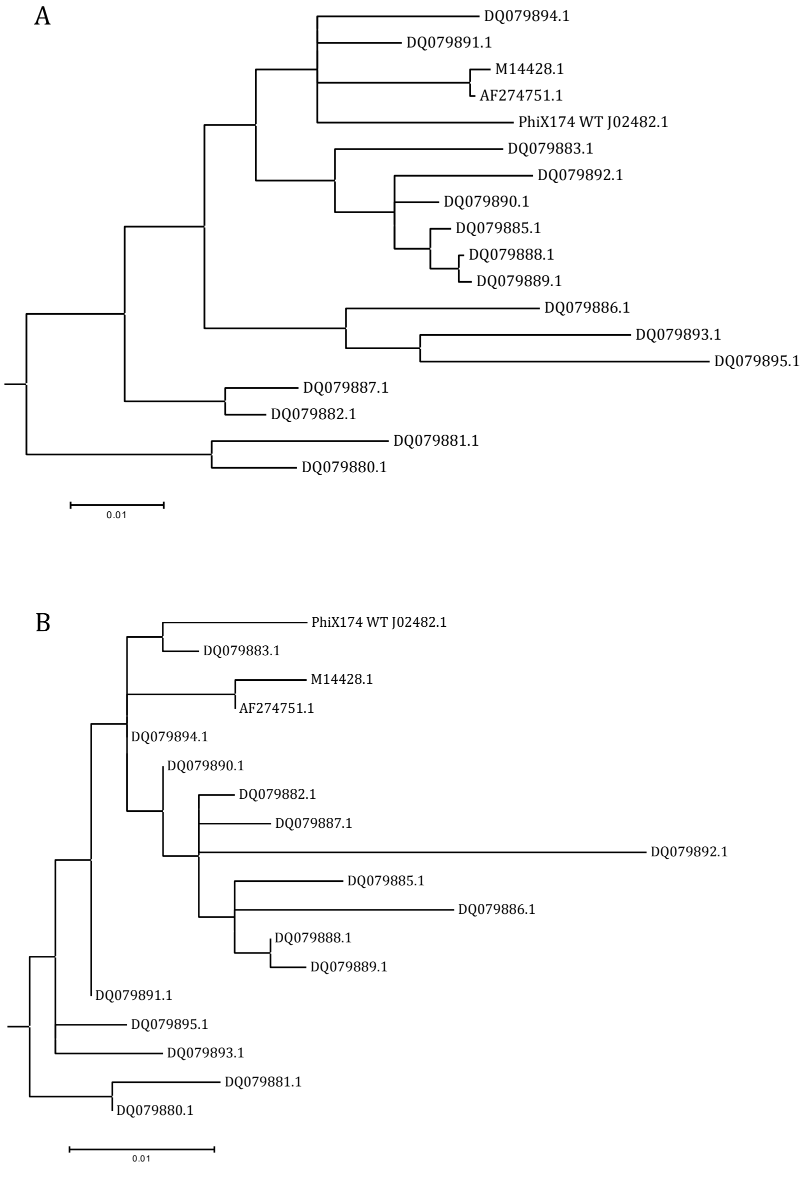

Figure 1: Consensus phylogenetic of the $\phi X174$ coat protein using MrBayes for: (A) Nucleotide and (B) amino acid datasets. The trees show only the ingroup sequences, although in the calculations the same outgroups as in Fig. 1 of the main text were used.
