# Supplementary material for: Evolutionary interplay between structure, energy and epistasis in the coat protein of the ϕX174 phage family
Source: J R Soc Interface. 2017 Jan;14(126):20160139. doi: 10.1098/rsif.2016.0139 (PMC5310724; doi:10.1098/rsif.2016.0139)
Supplement: Statistical methods for estimating epistasis [file rsif20160139supp2.pdf]

Evolutionary interplay between structure, energy, and epistasis in the coat protein of the $\phi X174$ phage family.

Electronic Supplementary Material 2: Statistical methods for estimating epistasis.

Rodrigo A.F. Redondo, Harold P. de Vladar,
Tomasz Włodarski and Jonathan P. Bollback

In this supplementary information we first present details on our calculations of the t-test employed to detect epistatic haplotypes, we report on the calculation of statistical epistasis.

1 T-test for structural epistasis

First, we consider that the average over samples of the free energy of a given haplotype $\mathcal{H}$ is

$$\overline{\Delta\Delta\mathrm{G}}_{\mathcal{H}} = \sum_{k\in\mathcal{H}} \overline{\Delta\Delta\mathrm{G}}_k + \epsilon_{\mathcal{H}} \quad (1)$$

where $\overline{\Delta\Delta\mathrm{G}}_k$ are the means of the additive values (single mutants), and $\overline{\Delta\Delta\mathrm{G}}_{\mathcal{H}}$ the mean of the multiple mutant. Thus $\epsilon_{\mathcal{H}}$ is the average epistatic value.

The test is an extension of the standard t-test for comparing samples. In this case we want to test the null hypothesis H_0 that epistasis, i.e.

$$\epsilon_{\mathcal{H}} = \overline{\Delta\Delta\mathrm{G}}_{\mathcal{H}} - \sum_{k\in\mathcal{H}} \overline{\Delta\Delta\mathrm{G}}_k \ , \quad (2)$$

is significantly different than zero. For this purpose we define the standard deviation of the pooled sample

$$S_{\mathcal{H}} = \sqrt{\frac{s^2_{\mathcal{H}}}{n_{\mathcal{H}}} + \sum_{k\in\mathcal{H}} \frac{s^2_k}{n_k}} \ , \quad (3)$$

where the s^2_k are the sample variances of $\Delta\Delta\mathrm{G}_k$.

In order to perform the t-test we also require the degrees of freedom $df_{\mathcal{H}}$. This is approximated with the Welch-Satterthwaite formula, which reads

$$df_{\mathcal{H}} = \frac{\left(\frac{s_{\mathcal{H}}^2}{n_{\mathcal{H}}} + \sum_{k\in\mathcal{H}} \frac{s_k^2}{n_k}\right)^2}{\frac{1}{n_{\mathcal{H}}-1}\left(\frac{s_{\mathcal{H}}^2}{n_{\mathcal{H}}}\right)^2 + \sum_{k\in\mathcal{H}} \frac{1}{n_k-1}\left(\frac{s_k^2}{n_k}\right)^2} \ . \tag{4}$$

Bonferroni correction

We need to apply the correction for multiple hypotheses tested on the same data. However, this number is not homogeneous across all tests because there are independent data sets used.

Lets start with the simplest case. That is with the haplotype that has all indexes {1,2,3,4,5,6,7,8}. Since all indexes are involved, all pairs, triplets, etc. are considered. We of course start count a 2 since there are no tests involving only one index. We have to add all unordered pairs, all unordered triplets, and so on. This comes down to the number of subsets minus the sets with individual elements (minus the empty set). Namely,

$$m_8 = 2^8 - 8 - 1 = 2^8 - 2^3 - 1 = 2^3\left(2^5 - 1\right) - 1$$

For haplotypes fixing a set of k indexes, we need to count the number of subsets that have at least one of the indices. Note that this is more simply calculated as the number of subsets of the remaining indices. There are $n-k$ unused indices of which there are 2^{n-k} subsets. Hence, the number of subsets that have at least one used index are $2^n - 2^{n-k}$. From this we finally subtract the number of sets with only one element, of which there are exactly k. Thus

$$m_k = 2^n - 2^{n-k} - k \ . \tag{5}$$

This clearly matches the calculation above for $n = k = 8$.

The corrected significance for each test is therefore

$$\alpha^* = \alpha \big/ \left(2^n - 2^{n-k} - k\right) \tag{6}$$

where α is the base significance. Figure 1 shows the corrected significance for the current data and for a base significance of α=0.05.

All the above functions were tested and implemented in *Mathematica* 10.0.1.0.

Figure 2 shows the results of the T test for our data set.

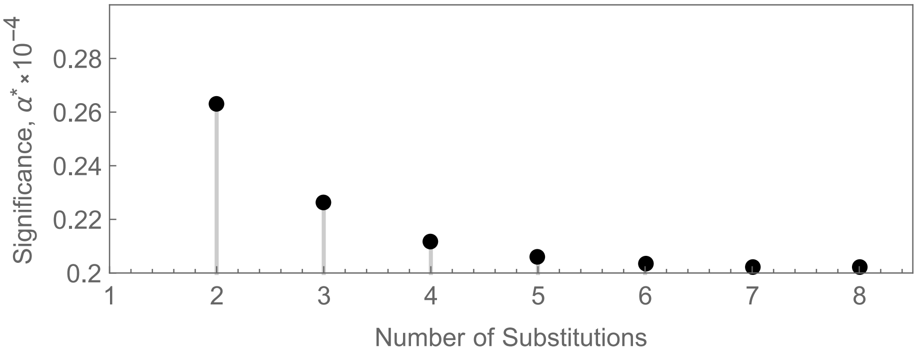

Figure 1: Bonferroni correction for different haplotype numbers in our structured data set.

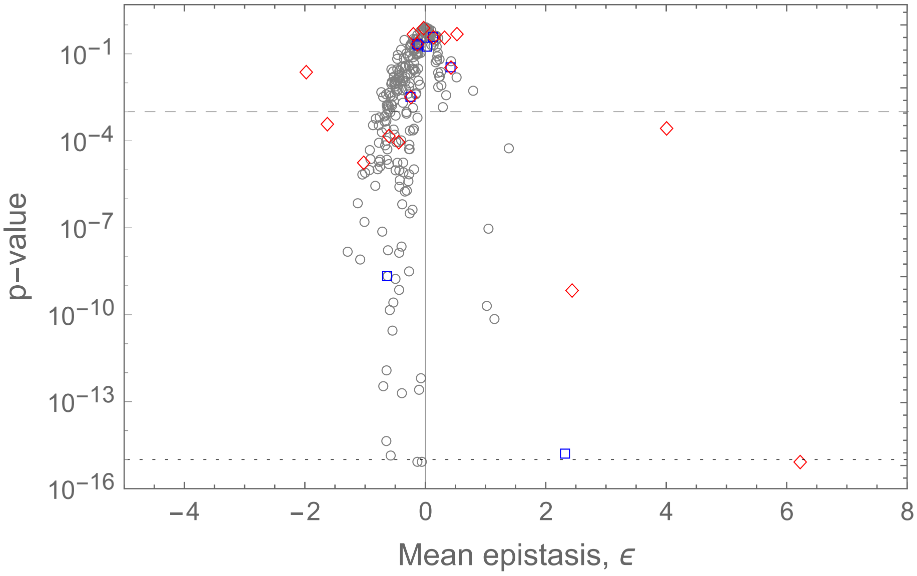

Figure 2: Resulting p-values for our data of free energies $\Delta\Delta G$ against the mean epistatic values ϵ. Dashed line $p = 1/256 \simeq 0.002$. Dotted line: $p = 10^{-15}$; $p-$values below this number are clipped in this figure. Grey rings: ancestral haplotypes; blue squares: internal nodes (other than ancestral); red diamonds: extant species.

2 Statistical epistasis

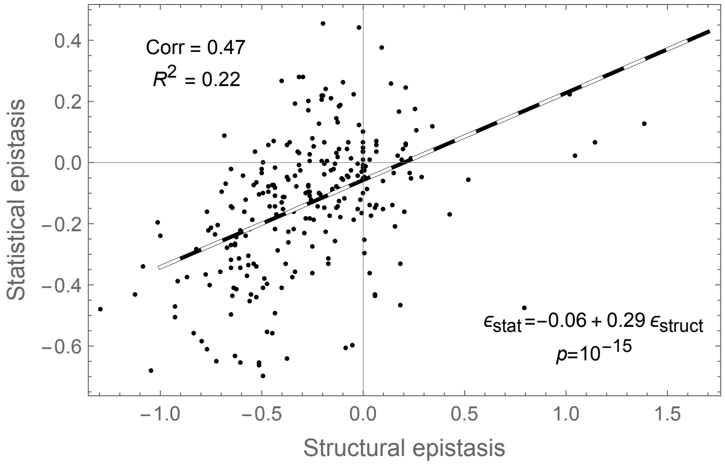

Figure 3: Correlation between statistical and structural epistasis in the ancestors (computed from independent sets of simulations). Dashed line: linear regression between the two measures. Each point is an average over at least 15 replicas.
