# Supplementary material for: Evolutionary interplay between structure, energy and epistasis in the coat protein of the ϕX174 phage family
Source: J R Soc Interface. 2017 Jan;14(126):20160139. doi: 10.1098/rsif.2016.0139 (PMC5310724; doi:10.1098/rsif.2016.0139)
Supplement: Calculations and comparisons with Rosetta [file rsif20160139supp3.pdf]

Evolutionary interplay between structure, energy, and epistasis in the coat protein of the $\phi X174$ phage family.

Electronic Supplementary Material 3: Calculations and comparisons with Rosetta.

Rodrigo A.F. Redondo, Harold P. de Vladar,
Tomasz Włodarski and Jonathan P. Bollback

In this supplementary file we summarise the methods and results with the package for structural analysis Rosetta. Although qualitatively the results differ between Rosetta and FoldX, they are consisten with each other qualitatively.

1 Calculation of $\Delta\Delta G$

We calculated the free energy change for the 256 ancestral haplotypes using the ART. As with FoldX, we only considered changes of free energy due to amino acid side chain reconfigurations while keeping the backbone fixed. Unlike with FoldX, we used only one copy of the coat protein and not the complete fragment because of the required limiting computational resources. In all simulations we employed 15 replicas and collected only the averaged $\Delta\Delta G$ values.

The distribution of free energy changes $\Delta\Delta G$ has a mean of -0.08 kcal/mol and variance of 0.79. Compared to that of FoldX, this distribution is significantly narrower, but the difference in means in non-significant. As with FoldX, Rosetta's results do not show any trend between the number of substitutions and free energy change $\Delta\Delta G$ (see main text).

2 Structural epistasis.

We computed the mean epistatic values as explained in the main text. That is, the difference between the $\Delta\Delta G$ and the additive $\Delta\Delta G_{add}$ of an haplotype. We did not perform a T-test as with FoldX because we only have the average values (only one point per haplotype) and thus lack statistical power. Figure 1A shows the distribution of mean epistasis from the Rosetta calculations, which has a mean value of -0.13 and a variance of 1.8. (Note that this distribution of epistasis is wider than that of FoldX, even though the free energy distributions follow

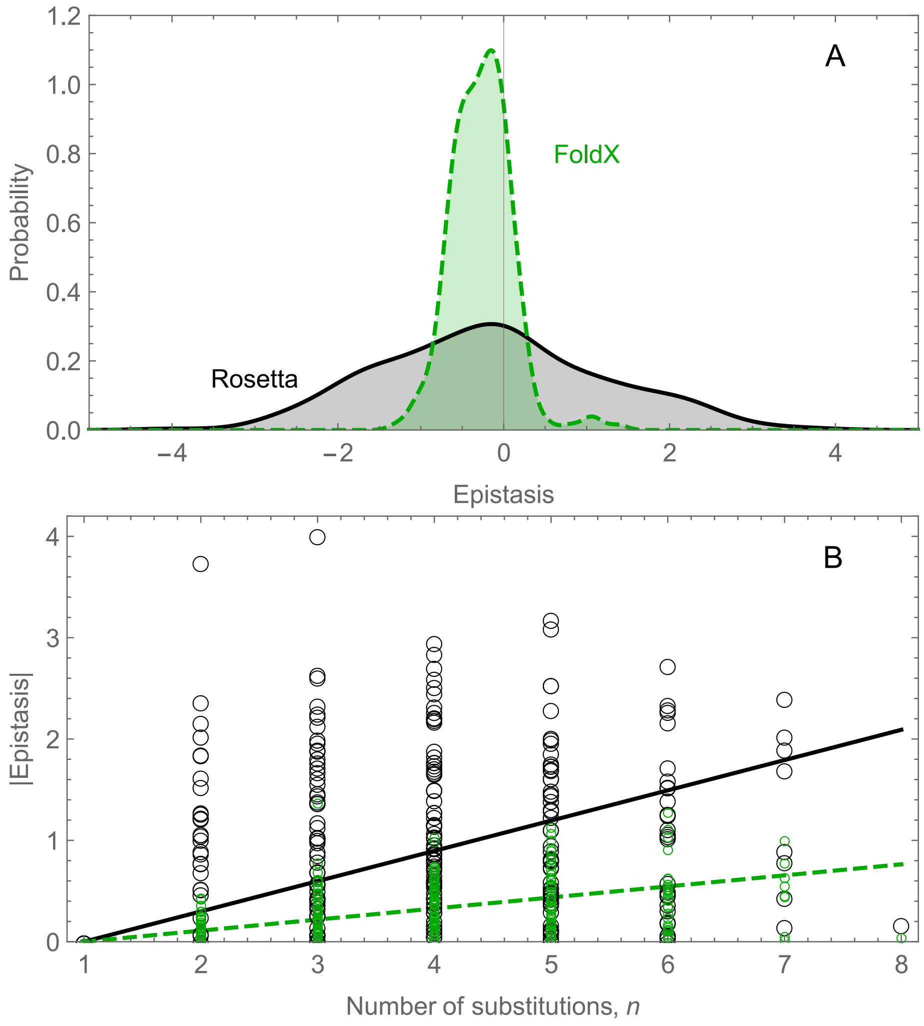

Figure 1: (A) Histograms of ancestral epistatic values from Rosetta (black solid line; mean=-0.13; variance=1.8) and from FoldX (green dashed line; mean=-0.28 kcal/mol; variance=0.14. Same as in Fig. 7 in the main text; shown as a reference). (B) Relationship between epistasis and the number of AA substitutions (n). Black bullets: Rosetta; small green bullets: FoldX. The lines are minimum square fits for the function $\epsilon = a(n-1)$. For Rosetta (black line) $\hat{a}_R = 0.30$ and for FoldX $\hat{a}_F = 0.11$.

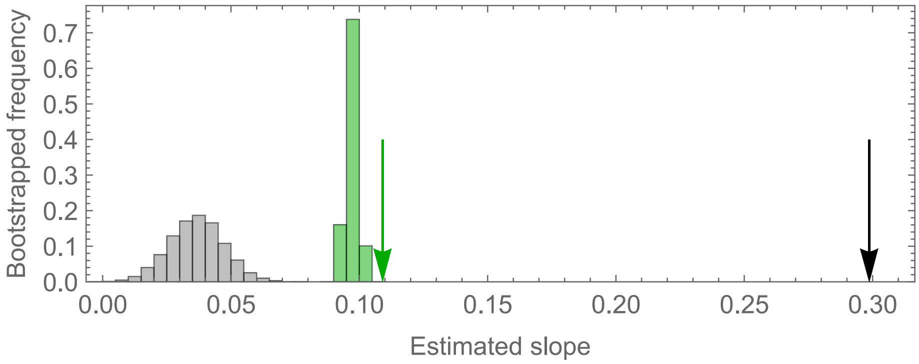

Figure 2: Bootstrapped slopes of epistasis vs. substitution number under randomisation test. Black: Rosetta, green: FoldX. Arrows: slopes from data (as in Fig. 1B

the contrary pattern.) However, the difference in the means of the distribution of epistasis in Rosetta and in FoldX is non-significant ($p = 0.3$), and the null hypothesis that they are equal cannot be rejected.

Both Rosetta and FoldX give consistent results regarding the trend of the strength of the epistatic values against the number of substitutions (Fig. 1A). To test the significance of the slopes we performed a bootstrap analysis where we randomised the values of epistasis in relation to the number of their constituting substitutions. Then for each randomised sample we computed the slope a of the function $\epsilon = a(n-1)$ (so that $\epsilon = 0$ for $n = 1$). From this calculation we obtained an empirical histogram of slopes. In other words, we tested against the distribution of slopes under the null hypothesis that the variance in epistasis is independent of the substitution number. We performed 10000 replicas for each test (one for Rosetta and one for FoldX) and in both cases we obtained $p < 10^{-5}$, rejecting the null hypothesis (Fig. 2). Thus, we conclude that the regression slopes are significant using both methods, Rosetta and FoldX.

3 High order structural epistasis

In a similar way as with FoldX (see main text and ESM3) deduced the distribution of high order epistasis from the $\Delta\Delta G$ values of Rosetta. Figure 3 shows that, as with FoldX, there is a notable mass of high order epistasis of positive mean (=2.0) and of large variance (=11.0). Because both distributions (total and high order epistasis) are derived from the same data, we do not perform any statistical analysis regarding their difference in means and variances. However, there is no statistical difference in the mean of the distributions of high order epistasis based on Rosseta's and FoldX's results. This is supporting evidence for the pervasiveness of high-order epistasis.

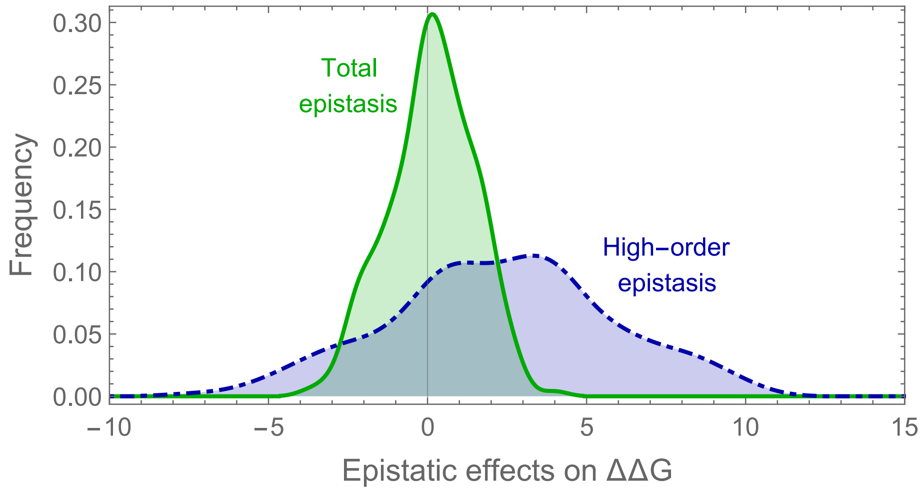

Figure 3: Histograms of total and high-order structural epistasis in the ancestors, estimated from Rosetta calculations. Solid green line: total epistasis (same as Fig. 1A, shown for reference); dot-dashed blue line: high-order epistasis (mean=2.0 kcal/mol; variance=11.0).
