# Supplementary material for: Evolutionary interplay between structure, energy and epistasis in the coat protein of the ϕX174 phage family
Source: J R Soc Interface. 2017 Jan;14(126):20160139. doi: 10.1098/rsif.2016.0139 (PMC5310724; doi:10.1098/rsif.2016.0139)
Supplement: Extended description of experimental procedures and results [file rsif20160139supp4.pdf]

Evolutionary interplay between structure, energy, and epistasis in the coat protein of the $\phi X174$ phage family.

Electronic Supplementary Material 4: Extended description of experimental procedures and results.

Rodrigo A.F. Redondo, Harold P. de Vladar, Tomasz Włodarski and Jonathan P. Bollback

To conduct the fitness assays comparing the ancestral polymorphisms on the coat protein gene F we synthesized each of the eight single variants (K83Q, T92S, P141A, E150Q, Q153E, Q182L, S339A, A361V), the ART and the AT_8.

Synthetic sequences of ancestral versions of gene F were obtained from Epoch Life Sciences Inc., for each of the eight single variants (K83Q, T92S, P141A, E150Q, Q153E, Q182L, S339A, A361V), for the ART and the AT_8. These sequences were cloned into the double stranded form (RF1) of the wild type ϕX174 (DSM4497, Sinsheimer/Sanger strain) replacing the SS gene F, and transformed into *E. coli* C strain (DSM13127).

To increase the titres and obtain mutant phage stocks, transformed *E. coli* were mixed with non-transformed bacteria, grown for 2 hours at 37°C. The lysate was then centrifuged at 4°C (10,000g for 5 minutes), filtered by a 0.22μM syringe-filter membrane and stored in 20% glycerol at -80°C.

The titre of the ϕX174 mutants stocks was determined by soft agar overlay method. Early exponential growth phase ($\sim 10^7$ cfu/mL) *E. coli* C cells and L.B. soft agar (0.7%) media, supplemented with 5mM of both $CaCl_2$ and $MgCl_2$, were mixed with serial dilutions of the stocks, plated on LB agar (1.5%), and incubated overnight at 37°C. Plaque counting for each sample (mutants and the SS) were done in 10 replicates with 2 plates/dilution.

To reduce measurement variance we performed structured experiments essaying each mutant paired with both the ART and of the SS. The data in Fig. 10 are averages over relative fitness measurements of these paired experiments, $W_i = \left\langle \frac{r_i}{r_o} \right\rangle$.

The experiments always included two replicates of the mutant being essayed plus both the SS and ART. Phages were diluted to [$\sim 10^3$ pfu/μL] based on the stock titre. 100μL of the replicates were mixed with 100μL of early-exponential

growth phase *E. coli* C (M.O.I = 0.0001) in 3mL of LB media.

To obtain initial titres at t_0, for each replicate one aliquot of 100μL was immediately plated along with two step-dilutions from a second 100μL aliquot (see titration above). The remaining mixture was incubated at 37°C for 1 hour following purification (as for phage stocks), an aliquot of 100μL was taken to obtain the t_{60} titre, diluted (3 $\times$ 10-fold steps) and plated (2 replicates per dilution).

Each paired experiment was performed twice on different days. The experiments were performed using a double blind design, only revealing the identity of the assays once the experiments were completed. Each mutant had a total of between 16-24 replicates depending on the number of countable plaques on the plates (SS and ART had 32-48 replicates), this experimental design accounts for variance in dilution, plating and handling during the experiments.

Further Experimental results

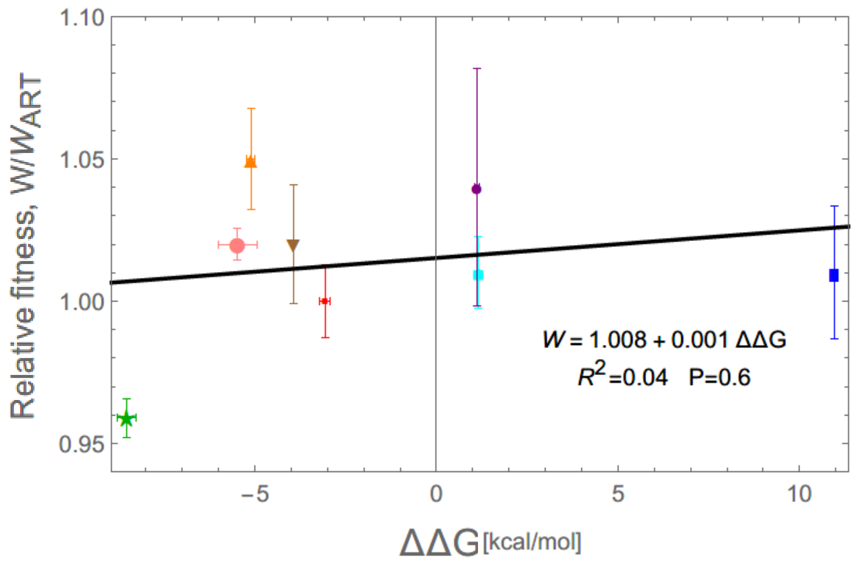

Figure 1: Relation between relative fitness and $\Delta\Delta G_{FX}$. Small red square: K83Q, magenta square: T92S, blue rectangle: P141A, green star: Q153E (consensus), orange upright triangle: Q182L, brown downwards triangle: S339A, small purple bullet: A361V, large grey bullet: AT_8.
