# Supplementary material for: Evolutionary interplay between structure, energy and epistasis in the coat protein of the ϕX174 phage family
Source: J R Soc Interface. 2017 Jan;14(126):20160139. doi: 10.1098/rsif.2016.0139 (PMC5310724; doi:10.1098/rsif.2016.0139)
Supplement: Inference of the fitness landscape [file rsif20160139supp5.pdf]

Evolutionary interplay between structure, energy, and epistasis in the coat protein of the $\phi X174$ phage family.

Electronic Supplementary Material 5: Inference of the fitness landscape.

Rodrigo A.F. Redondo, Harold P. de Vladar,
Tomasz Włodarski and Jonathan P. Bollback

Here we show by using theoretical and empirical arguments that the mutational variance generated by molecular substitutions is consistent with the stabilising selection scenario. Furthermore, this explains the distribution of free energies of the extant species. We also estimate the selective coefficient and the optimum value of the fitness landscape.

First of all, recall that selection is defined by the relationship

$$p_s(x) = p(x)w(x)\,, \tag{1}$$

where $p(x)$ is the frequency of the trait with value x (e.g. $x \equiv \Delta\Delta G$), and the subscript s indicated the distribution after selection; $w(x)$ is the relative fitness in the population of the trait value x. This implies that for every value of x, the fitness can be estimated by

$$w(x) = \frac{p_s(x)}{p(x)}\ . \tag{2}$$

Our task is to have an estimate of $w(x)$, which in turn implies having an estimate of the distributions $p_s(x)$ and $p(x)$. If we asume that (a) the ancestral population has a similar distribution to the inferred distribution of ancestral haplotypes and (b) that this distribution is proximately the equilibrium distribution then we can take it as a sample for p_s. We take as p, the distribution before selection, as the distribution generated by mutational effects (see Results and Methods sections). However, for our calculations involving p we only consider single substitutions. To facilitate our calculations we estimate these distributions using a gaussian kernel estimator. That is,

$$\hat{p}(x) = \frac{1}{nh}\sum_{i=1}^{n}\phi\left(\frac{x - x_i}{h}\right)\,, \tag{3}$$

where n is the number of histogram bins, h is the 'bandwidth', which accounts for the smoothing of the underlying histogram, and $\phi(x) = \exp(-x^2/2)/\sqrt{2\pi}$ is the gaussian kernel. The resulting smoothed distributions for the complete data (i.e. including multiple substitutions) are shown in the inset of the Fig. 5A in the main text with bandwidth $h = 0.1$.

Now se simply take the ratio $r(x) = \hat{p}_s(x)/\hat{p}(x)$, which is an empirical estimator of the fitness landscape $\hat{w}(x)$ (Fig. of the main text, dashed lines, $h = 0.3$).

We now assume that gaussian stabilising selection is acting, so that

$$w(x) = c \exp\left[-\frac{S}{2}(x - x_{opt})^2\right] . \tag{4}$$

Here, c is an arbitrary constant, S is the selective value, and x_{opt} is the optimal phenotype, in our case, the optimal free energy value. This parametrisation of the fitness landscape in terms of a gaussian function is standard in the literature of population genetics, largely for mathematical convenience. Although other forms are possible, the gaussian form captures the general relevant features of stabilising selection. Since we are interested only in rough estimates, we adhere to this standard for easiness.

Because the constant c is arbitrary (since fitness is always with respect to the mean fitness of the population), we simply take a normalised version of w, so that $c = \sqrt{S/2\pi}$. This calls also to normalise the ratio $r(x)$, which we perform as a numerical integration. Because $w(x)$ has a gaussian form, the integral of its first and second central moments (mean and variance respectively) are equated to the first and central moments of $r(x)$. Using $h = 0.3$, the mean gives us the optimum trait value $\Delta\Delta G_{opt} = -8.82$ kcal/mol, and the inverse of the variance gives the selective values $S \simeq 0.0071$. It is remarkable that this simple estimation of the optimum trait value coincides almost perfectly with the peak of the empirical distribution of free energies of the extant species (Fig. 5A in the main text), even though this data was not used in the estimation.

Figure 11 in the main text shows the empirical ratio and the inferred stabilising selection landscape. Clearly, this hasty calculation results in considerable deviations, obvious in the graphic. Yet, the gaussian landscape does reproduce well the general features, allowing for some robust inference.

As a final note, the ratio $r(x)$ is not too sensitive to the bandwidth h. There is little with the variation with h, where $\Delta\Delta G_{opt} \simeq -7.85$ kcal/mol and $S = 0.0080$ for $h = 0.1$. However, we favour the former estimate on the basis that it also matches the central mass of the distribution of free energies of the extant species.
